# Integrating radiomics features and CT semantic characteristics for predicting visceral pleural invasion in clinical stage Ia peripheral lung adenocarcinoma

**DOI:** 10.1007/s12672-025-02548-6

**Published:** 2025-05-16

**Authors:** Fengnian Zhao, Yunqing Zhao, Zhaoxiang Ye, Qingna Yan, Haoran Sun, Guiming Zhou

**Affiliations:** Department of Ultrasound, Tianjin Medical University General Hospital, Anshan Road, Heping District, Tianjin, 300052 China

**Keywords:** Computed tomography, Radiomics, Adenocarcinoma, Non-small cell lung cancer, Visceral pleural invasion

## Abstract

**Objectives:**

The aim of this study was to non-invasively predict the visceral pleural invasion (VPI) of peripheral lung adenocarcinoma (LA) highly associated with pleura of clinical stage Ia based on preoperative chest computed tomography (CT) scanning.

**Methods:**

A total of 537 patients diagnosed with clinical stage Ia LA underwent resection and were stratified into training and validation cohorts at a ratio of 7:3. Radiomics features were extracted using PyRadiomics software following tumor lesion segmentation and were subsequently filtered through spearman correlation analysis, minimum redundancy maximum relevance, and least absolute shrinkage and selection operator regression analysis. Univariate and multivariable logistic regression analyses were conducted to identify independent predictors. A predictive model was established with visual nomogram and independent sample validation, and evaluated in terms of area under the receiver operating characteristic curve (AUC).

**Results:**

The independent predictors of VPI were identified: pleural attachment (p < 0.001), pleural contact angle (p = 0.019) and Rad-score (p < 0.001). The combined model showed good calibration with an AUC of 0.843 (95% confidence intervals (CI 0.796, 0.882), in contrast to 0.757 (95% CI 0.724, 0.785; DeLong’s test P < 0.001) and 0.715 (95% CI 0.688, 0.746; DeLong’s test P < 0.001) when only radiomics or CT semantic features were utilized separately. For validation group, the accuracy of combined prediction model was reasonable with an AUC of 0.792 (95% CI 0.765, 0.824).

**Conclusion:**

Our predictive model, which integrated radiomics features of primary tumors and peritumoral CT semantic characteristics, offers a non-invasive method for evaluating VPI in patients with clinical stage Ia LA. Additionally, it provides prognostic information and supports surgeons in making more personalized treatment decisions.

**Supplementary Information:**

The online version contains supplementary material available at 10.1007/s12672-025-02548-6.

## Highlights

1. Peritumoral CT semantic characteristics were significantly associated with VPI in peripheral LA patients with clinical stage Ia, instead of the CT semantic features of primary tumor.
2. Radiomics analysis of the primary tumor has the potential to enhance the predictive accuracy of models, as evidenced by a significant increase in the AUC of the combined model.

**Supplementary Information:**

The online version contains supplementary material available at 10.1007/s12672-025-02548-6.

## Introduction

Lung cancer represents a significant proportion of cancer-related mortality worldwide, of which approximately 80–85% is non-small cell lung cancer (NSCLC), with lung adenocarcinoma (LA) being the predominant histological subtype [1, 2]. The past decade has witnessed significant progress in early detection through widespread implementation of low-dose thin-section computed tomography (CT) screening, leading to increased identification of small-volume lung cancers. Visceral pleural invasion (VPI), defined as tumor extension beyond the elastic layer with or without pleural surface involvement (while not extending to neighboring anatomical structures) [3], has been consistently recognized as a poor prognostic factor in NSCLC ≤ 3 cm for decades [4]. Specifically, VPI upstages T classification from T1 to T2, consequently advancing overall tumor staging from Ia to Ib according to the 8 th tumor lymph node metastasis (TNM) classification [5]. This staging alteration carries substantial clinical implications: the recommended surgical approach shifts from limited resection (segmentectomy) to radical lobectomy with systematic lymph node (LN) dissection when VPI is present in T1-stage tumors [6].

While chest CT remains the cornerstone non-invasive modality for preoperative evaluation of pulmonary lesions, reliable identification of VPI remains challenging radiologically, particularly for subpleural tumors. Recent advances in radiomics, a computational technique that extracts high-dimensional quantitative features through machine learning algorithms, have shown promise in NSCLC characterization [7]. Previous researches [8, 9] have indicated that VPI is rarely observed in pure ground glass nodules (GGNs) due to the limited ability to penetrate the thick elastic layer.

This dual-center retrospective study therefore focused on clinical stage Ia peripheral LA exhibiting either subpleural location or pleural indentation/tagging signs. We developed an integrative predictive model combining CT semantic characteristics with radiomics signatures, aiming to non-invasively preoperatively identify VPI and subsequently guide surgical decision-making regarding resection extent and nodal dissection requirements.

## Methods

### Patients

**Fig. 1:**
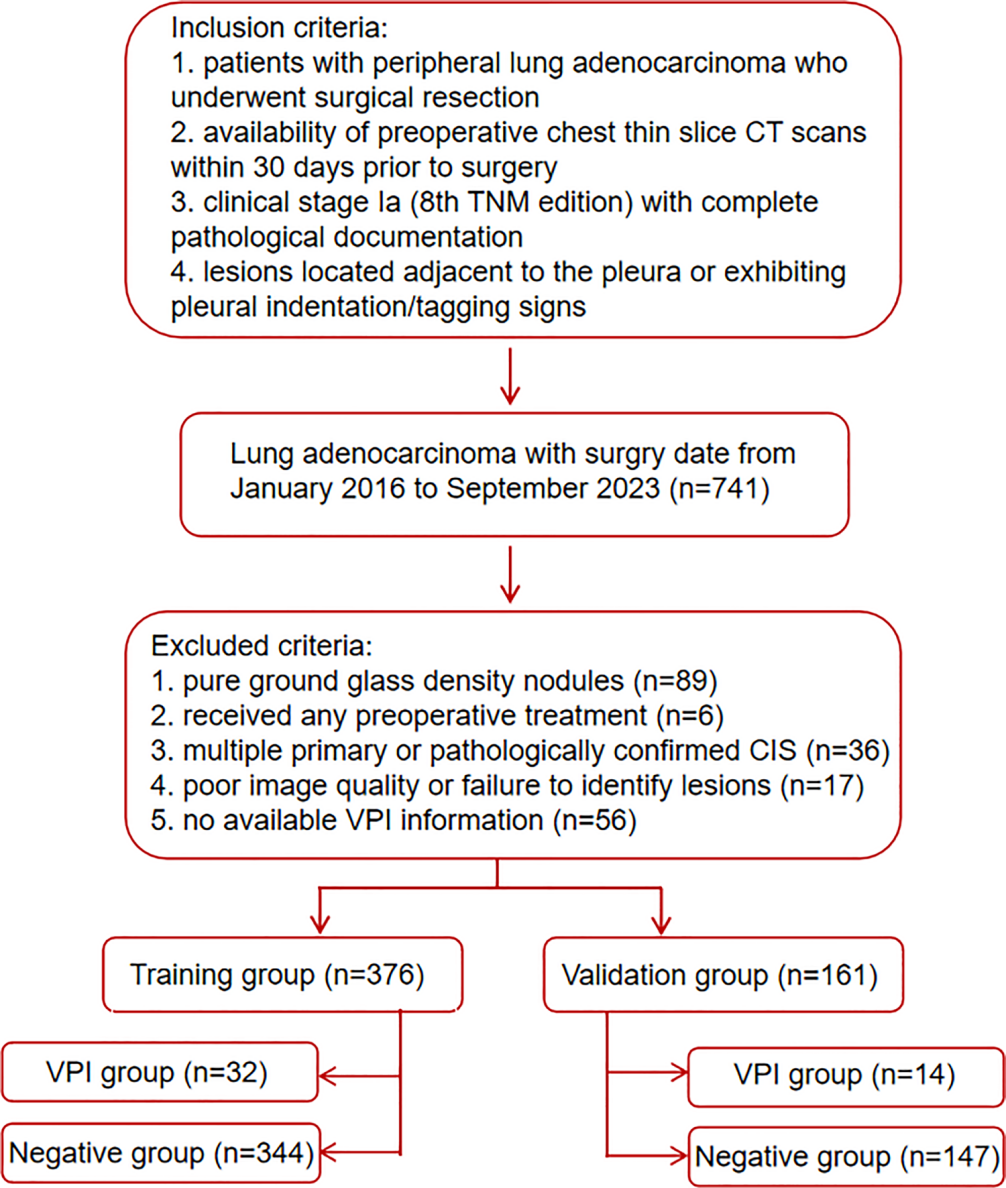
Flowchart of patients selection and exclusion. *CT* computed tomography, *CIS* carcinoma in situ, *VPI*,visceral pleural invasion

The study was conducted in accordance with the Declaration of Helsinki (as revised in 2013). The study was approved by the Medical Research Ethics Committee and the Institutional Review Board of Tianjin Medical University General Hospital (No. IRB2024-KY-240) and Tianjin Medical University Cancer Institute and Hospital (No. Ek2021067), and individual consent for this retrospective analysis was waived. Initially, a total of 741 patients were enrolled with surgery date from January 2016 to September 2023 according to the following inclusion criteria: (a) patients with peripheral LA who underwent surgical resection; (b) availability of preoperative chest thin slice CT scans including both non-contrast and contrast-enhanced series within 30 days prior to surgery; (c) clinical stage Ia (8 th TNM edition) with complete pathological documentation; and (d) subpleural lesions defined as tumor-pleura distance ≤ 2 cm or presence of pleural indentation/tagging signs. We excluded patients who had received preoperative treatment (n = 6), lesions with a pure ground glass density (n = 89), images of poor quality or inability to identify lesions (n = 17), no available VPI information (n = 56), as well as cases of pathologically confirmed multiple primary lung cancer and carcinoma in situ (n = 36) (Fig. 1). The final cohort included 537 patients (385 from Tianjin Medical University Cancer Hospital; 152 from Tianjin Medical University General Hospital), randomly allocated into training (70%, n = 376) and validation (30%, n = 161) cohorts using computer-generated randomization.

Demographic and clinical parameters (age, sex, smoking history, familial cancer predisposition, and driver gene mutations) were retrieved from institutional databases. Histopathological classification followed the 2015 World Health Organization guidelines [10], with histological subtypes components > 5% documented on the form according to their proportion. Risk stratification was implemented as: low-risk (minimally invasive adenocarcinoma); intermediate-risk (acinar/lepidic/papillary predominant invasive LA) and high-risk (invasive mucinous/colloid/fetal/enteric LA or micropapillary/solid predominant tumors). TNM staging was conducted following the guidelines of the 8 th edition criteria published by the Union for International Cancer Control and the American Joint Committee on Cancer [5].

### Evaluation of clinical stage Ia and VPI

Preoperative staging incorporated chest CT (tumor dimensions, LN evaluation) supplemented by whole-abdomen CT, brain magnetic resonance imaging, bone scintigraphy, or positron emission tomography to exclude distant metastases. LNs were considered negative (cN0) when short-axis diameters < 10 mm on axial CT images. Elastic van Gieson staining was utilized to evaluate the presence of VPI by a specialized pathologist (Dr. Yan Q., 30 year oncopathology experience), who was blinded to the patients’ clinical and radiological data. Pleural invasion levels (PL) were categorized as: PL0, no pleural invasion, PL1, tumor invasion of the visceral pleural elastic layer without reaching the surface of the visceral pleura, PL2, tumor invasion of the visceral pleural surface, and PL3, tumor invasion of the parietal pleura or chest wall [11]. Per established criteria [3], cases were dichotomized into VPI-negative (PL0) and VPI-positive (PL1-2) groups for analytical purposes.

### CT scanning protocol

Chest CT examinations were performed using five multidetector CT systems of three types: Lightspeed16, GE Healthcare, Milwaukee, WI, USA; Somatom Sensation 64, Siemens, Erlangen, Germany; Discovery CT750 HD, GE Healthcare. The scanning parameters were: (a) 120 kVp with the automatic regulation of the tube current and 1.5 mm reconstruction thickness and intervals for the 64-detector scanner and (b) 120 kVp, 150–200 mAs, and 1.25 mm reconstruction thickness intervals for the other two types of scanners.

### CT image interpretation and preprocessing

Two clinical radiologists, one with 5 years’ and the other with 8 years’ expertise in thoracic malignancy CT imaging, independently analyzed the CT scans, following a blinded training protocol with clinical and pathological data withheld. Any discrepancies in image interpretation will be guided by a senior radiologist with over 35 years of expertise to reach a consensus. Detailed CT semantic descriptors and scoring criteria for both primary tumors and peritumoral regions are presented in Table 1. All image analyses were performed using standardized window settings: lung window (width 1500 HU, level − 600 HU) and mediastinal window (width 350 HU, level 40 HU). The evaluation of CT descriptors was conducted on multi-planar reconstructed images and documented using a standardized scoring sheet. All CT images underwent standardized preprocessing including: image resampling to 1 mm slice thickness using linear interpolation, followed by noise reduction through Gaussian filtering (σ = 0.5 mm).

**Table 1:** CT semantic features for lung adenocarcinoma

Characteristic	Definition	Scoring and definition
Maximum diameter	The greatest dimension on the multiplanar reconstructed images with a lung window	cm
Consolidation diameter	The greatest dimension on the multiplanar reconstructed images with a mediastinal window	cm
TDR	1—consolidation diameter/maximum diameter	
Contour	The overall shape of roundness	1, round or oval; 2, somewhat irregular; 3, irregular
Lobulation	A wavy or scalloped configuration of tumor’s surface	0, absence; 1, presence
Spiculation	Lines radiating from the margins of the tumor	0, absence; 1, presence
Texture	Solid or GGO	1, mixed GGO with solid part < 50%; 2, mixed GGO with solid part > 50%; 3, solid
Calcification	Any patterns of calcification in the tumor	0, absence; 1, presence
Air bronchogram	Tubelike or branched air structure within the tumor	0, absence; 1, presence
Bubble-like lucency	Air space in the tumor with diameter ≤ 5 mm at the time of diagnosis prior to biopsy or treatment	0, absence; 1, presence
Cavity	Air space in the tumor with diameter > 5 mm at the time of diagnosis prior to biopsy or treatment	0, absence; 1, presence
Pleural indentation	indentation of the pleura toward the tumor	0, absence; 1, presence
Pleural attachment	Tumor attaches to the fissure/Pleura	0, absence; 1, presence
Pleural contact angle	The angle of the solid components of tumor contacting to the surface of pleura	0, non-contact; 1, angle small than 90°; 2, angle not small than 90°
Pleural contact length	The maximum length of the pleural contact curve for solid components	cm
DLP	The closest distance from the edge of lesions to the adjacent pleura	cm
Bronchovascular bundle thickening	Convergence of vessels to the tumor	0, no significant thickening; 1, obvious thickening
Obstructive change	Consolidation shadow caused by obstructive pneumonia or atelectasis at the edge of tumor	0, absence; 1, presence
Pleural effusion of tumor side	Pleural effusion seen in the tumor side of the thoracic cavity	0, absence; 1, presence
Pleural effusion of non-tumor side	Pleural effusion seen in the nontumor side of the thoracic cavity	0, absence; 1, presence

CT, computed tomography; TDR, tumor shadow disappear rate; GGO, ground-glass opacity; DLP, distance from the lesions to the adjacent pleura.

### Tumor segmentation and features extraction

Tumor segmentation was carried out independently by two radiologists utilizing ITK-SNAP software (version 3.6.0), using the manual method of drawing regions of interest (ROI) on CT images at the lung window. The radiologists were informed of the tumors'location but remained blinded to additional pertinent information. Radiomics features were automatically extracted using PyRadiomics (version 3.0), an open-source software (http://www.radiomics.io/pyradiomics.html) [12]. A total of 1316 radiomics features were extracted from the 3D ROIs, including first-order statistics (n = 108), shape-based features (n = 14), gray level cooccurrence matrix (n = 144), gray level dependence matrix (GLDM, n = 84), gray level run-length matrix (n = 96), gray level size zone matrix (GLSZM, n = 96), neighboring gray tone difference matrix (n = 30) and higher-order wavelet features (n = 744).

### Feature selection and establishment of radiomics signature

ComBat calibration [13] was preformed to remove batch effects between different scanners (Table S1). Then, the radiomics parameters extracted by two radiologists were averaged after standardizing using the Z-score method:

$$Z = {{\left( {{\text{X}} - \mu } \right)} \mathord{\left/ {\vphantom {{\left( {{\text{X}} - \mu } \right)} \sigma }} \right. \kern-0pt} \sigma },$$

where X is the original eigenvalue, μ is the mean eigenvalue, and σ is the standard deviation. Spearman pairwise correlation analysis was performed to exclude features with absolute correlation coefficients > 0.9, effectively removing redundant variables. Subsequently, the minimum redundancy maximum relevance (mRMR) algorithm identified the top 100 most informative features, which then underwent least absolute shrinkage and selection operator (LASSO) regression to determine the optimal feature subset for predicting VPI. These selected features were incorporated into a logistic regression model, with non-significant variables iteratively removed. The final radiomics score (Rad-score) was calculated using the formula:

$${\text{Rad - score}}\,{ = }\,{\text{b}}\,{ + }\,\sum {\left( {{\text{Ci}} \times {\text{Xi}}} \right)} ,$$

where b represents the intercept term, Xi denotes the standardized feature value, and Ci corresponds to the regression coefficient. Rad-score for each patient was calculated to assess the disparity between different groups.

### Statistical analysis

Statistical analyses were conducted using R software (version 4.3.0) and SPSS (version 26.0). Interobserver agreement was assessed using the ĸ index and Kendall coefficient of concordance. The non-parametric two-sample Wilcoxon test was utilized for ranked or continuous variables, while chi-square or Fisher's exact tests were employed for categorical variables in univariate analysis. Multivariate logistic regression analyses were conducted to evaluate the ability to identify VPI in various models. The predictive models were generated using the ten-fold cross-validation and the bootstrap method. The latter randomly generated a 90% sample of the data, which was repeated 1000 times and the results were averaged with 95% bootstrap confidence intervals (CI). Model discrimination was evaluated through receiver operating characteristic (ROC) curve analysis with DeLong's test for area under the curve (AUC) comparisons, while calibration was verified using Hosmer–Lemeshow test and calibration curve. Clinical utility was quantified via decision curve analysis (DCA) across threshold probabilities. The final model was visualized as a nomogram, with results reported as mean AUC values and 95% CI derived from bootstrap resampling. P values < 0.05 were regarded as statistically significant.

## Results

### Inter-observer consistency analysis and patient demographics

Agreement among the two readers was good (Table S1 and S2). The intraclass correlation coefficient for maximum diameter, consolidation diameter, tumor shadow disappear rate, pleural contact length and distance from the lesions to pleura (DLP) was 0.92 (range, 0.90–0.94), 0.86 (range, 0.84–0.89), 0.88 (range, 0.86–0.90), 0.91 (range, 0.87–0.92) and 0.92 (range, 0.89–0.95), respectively. No significant difference of either feature was observed between training and validation group (Table S3).

### Correlation of VPI with clinical and CT semantic features in training group

32 patients in training group developed VPI as confirmed by postoperative pathology. Accordingly, the patients were categorized into VPI and negative groups. The association between clinical features with VPI was presented in Table 2. Significantly, Patients with epidermal growth factor receptor (EGFR) of wild-type [8/63 (12.7%) vs. 6/132 (4.5%)] developed VPI more frequently than EGFR mutant patients (odds ratio (OR) = 3.06, 95% CI 1.01, 9.22; p = 0.039). No significant association was noted for other clinical features.

**Table 2:** Association between clinical characteristics with VPI in training group

Variable	Negative Group	VPI Group	P Value	Univariate OR (95% CI)
Number	344	32		
Age (years)	59.60 (± 8.43)	58.16 (± 9.60)	0.635	
Sex				
Male	134	14	0.595	
Female	210	18		
Smoking history				
Yes	137	15	0.437	
No	207	17		
Family history				
Yes	79	8	0.794	
No	265	24		
Histological subtype				
Low risk	31	0	0.902	
Moderate risk	141	18		
High risk	172	14		
EGFR				
Mutation	126	6	0.039	Reference
Wild	55	8		3.06 (1.01, 9.22)
KRAS				
Mutation	9	1	1.000	
Wild	142	13		
ALK				
Positive	9	2	0.629	
Negative	124	12		

Data for age is mean ± standard deviation

VPI, visceral pleural invasion; OR, odds ratio; CI, confidence interval; EGFR, epidermal growth factor receptor; KRAS, kirsten rat sarcoma viral oncogene; ALK, anaplastic lymphoma kinase.

**Fig. 2:**
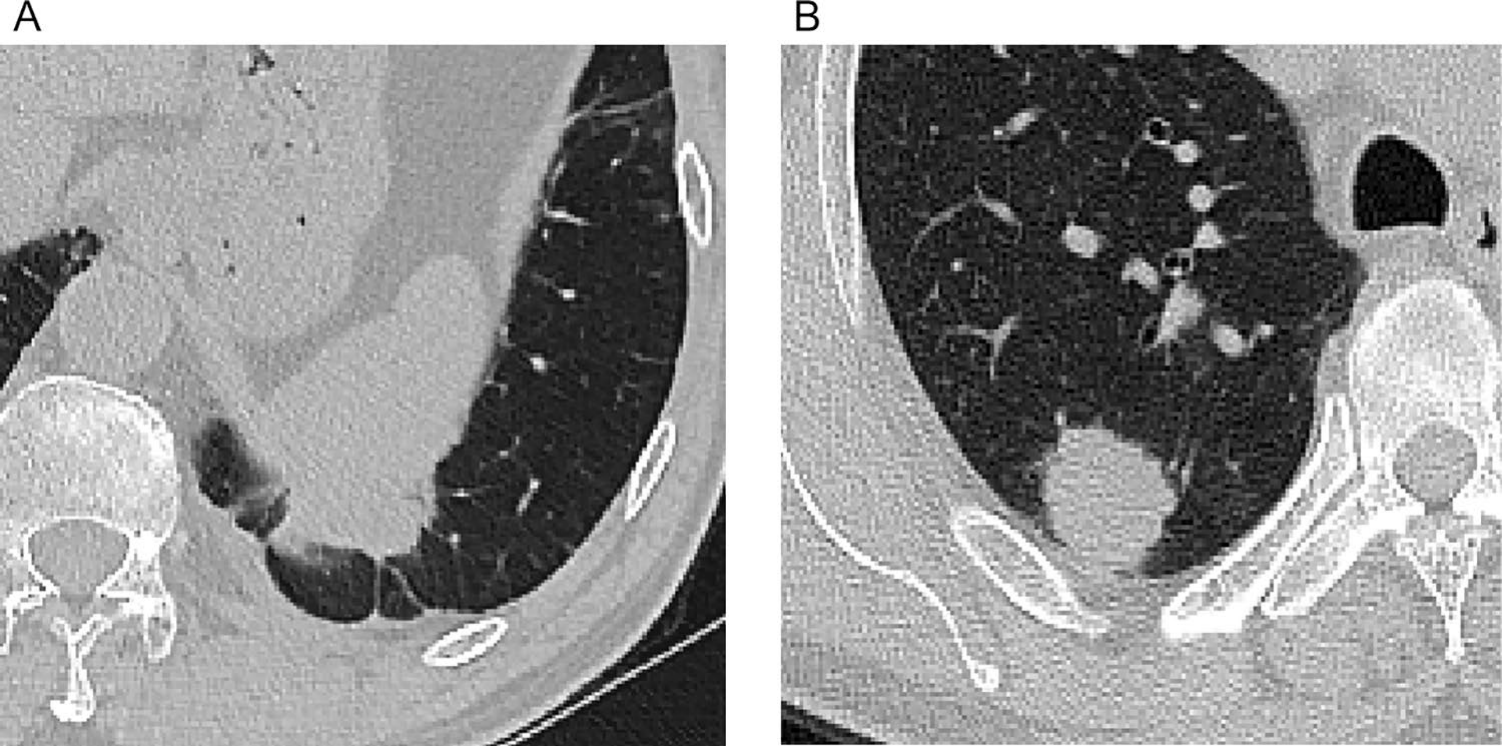
**A** In a case of lung adenocarcinoma with VPI, the CT scan lung window image reveals the tumor's positioning beneath the pleura, with pleural attachment and a long pleural contact length characterized by an obtuse angle of contact. **B** Conversely, in a case of lung adenocarcinoma without VPI, the CT scan lung window image depicts the tumor located beneath the pleura, with a shorter curve length and an acute angle of contact

Univariate analysis showed that tumors with smaller DLP (p < 0.001), larger pleural contact length (p < 0.001) and pleural contact angle (OR = 4.57, 95% CI 1.92, 10.84 for score 1; OR = 3.28, 95% CI 1.56, 6.87 for score 2; p < 0.001), and pleural attachment (OR = 4.57, 95% CI 1.92, 10.84; p < 0.001) were more likely to develop VPI (Table 3; Fig. 2). Additionally, the CT semantic features of the primary tumor did not show statistical significance (Table S4).

**Table 3:** Association between peritumoral semantic features with VPI in training group

Variable	Negative Group	VPI Group	P Value	Univariate OR(95% CI)
Number	344	32		
Pleural attachment				
0	190	7	< 0.001	Reference
1	154	25		4.57 (1.92, 10.84)
Pleural indentation				
0	104	8	0.536	
1	240	24		
Bronchovascular bundle thickening				
0	274	27	0.522	
1	70	5		
Obstructive change				
0	326	32	0.384	
1	18	0		
Pleural contact angle				
0	190	7	< 0.001	Reference
1	81	10		4.57 (1.92, 10.84)
2	73	15		3.28 (1.56, 6.87)
DLP	0.47 (± 0.66)	0.13 (± 0.28)	< 0.001	
Pleural contact length	1.29 (± 0.49)	1.67 (± 0.73)	< 0.001	
Pleural effusion of tumor side				
0	344	32	NA	
1	0	0		
Pleural effusion of non-tumor side				
0	343	32	1.000	
1	1	0		

Data for DLP and pleural contact length are mean ± standard deviation

VPI, visceral pleural invasion; OR, odds ratio; CI, confidence interval; DLP, distance from the lesions to the adjacent pleura; NA, not applicable.

### Screening and integration of radiomics features

**Fig. 3:**
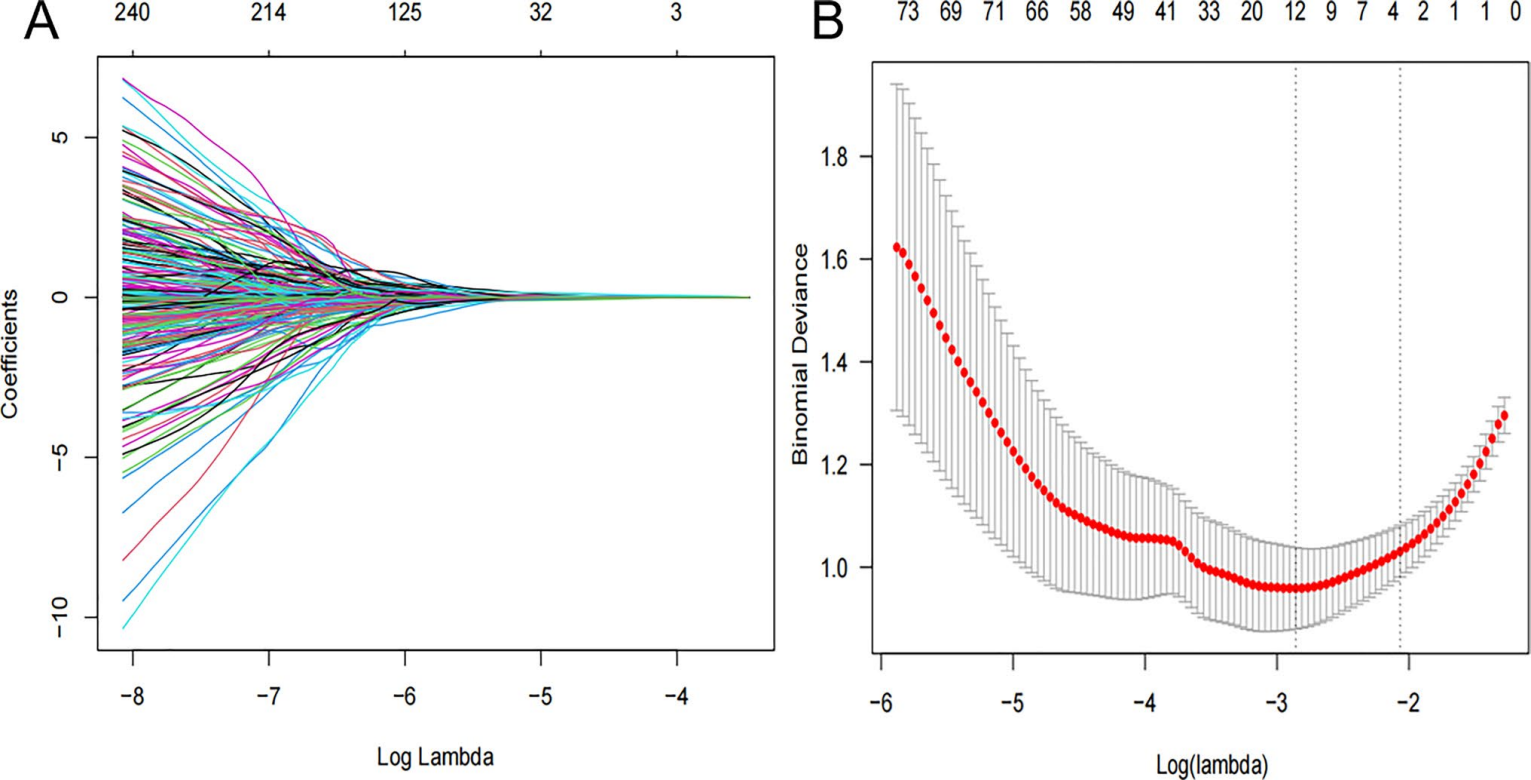
**A** Radiomics features screened by the LASSO regression model. The horizontal axis represents the log lambda, and the vertical axis represents the coefficient of each feature. **B** The mean squared error of radiomics features displayed by the Lasso regression analysis. Two vertical lines represent the lambda values when number of variables decreased to two lowest levels

A comprehensive analysis was conducted on a dataset comprising 1316 radiomics features extracted from the three-dimensional ROIs. Following Spearman correlation analysis and mRMR ranking, LASSO identified 12 non-redundant features (Table 4; Fig. 3). Subsequent backward elimination logistic regression excluded 7 non-significant variables, yielding 5 robust radiomics predictors. These were incorporated into a radiomics signature (Rad-score) weighted by their respective coefficients (Table 4). The Rad-score demonstrated significant discriminative capacity between VPI-positive (2.45 ± 1.67) and negative (−0.93 ± 1.83) cohorts (p < 0.001), indicating strong group separation.

**Table 4:** The radiomics features in rad-score formula* after the least absolute shrinkage and selection operator algorithm and logistic regression analysis

Radiomics Features	Significant predictors	
	P Value	Odds ratio (95% CI)
original_glszm_Large Area Low Gray Level Emphasis	0.005	1.76 (1.54, 2.13)
exponential_firstorder_Total Energy	< 0.001	2.32 (1.51, 3.24)
exponential_gldm_Small Dependence High Gray Level Emphasis	NA	
exponential_glrlm_Run Length Non Uniformity	NA	
square_gldm_Dependence Non Uniformity Normalized	0.033	1.11 (0.75, 1.63)
square_glszm_Small Area Low Gray Level Emphasis	NA	
square_ngtdm_Busyness	NA	
squareroot_firstorder_Skewness	NA	
wavelet-LLH_glcm_MCC	NA	
wavelet-LHL_firstorder_Skewness	< 0.001	0.30 (0.14, 0.75)
wavelet-HLL_glszm_Large Area Low Gray Level Emphasis	NA	
wavelet-LLL_gldm_Dependence Entropy	0.017	1.21 (0.86, 1.71)

Rad-score, radiomics score; CI, confidence interval; NA, not applicable (variables that were not included in the equation of multivariate logistic regression analysis with backward stepwise selection).

^*^Rad-score = −0.855 + 0.619 * exponential_firstorder_Total Energy + 0.428 * original_glszm_Large Area Low Gray Level Emphasis + 0.332 * square_gldm_Dependence Non Uniformity Normalized—0.508 * wavelet-LHL_firstorder_Skewness + 1.046 * wavelet-LLL_gldm_Dependence Entropy

### Predictive model and validation test

**Fig. 4:**
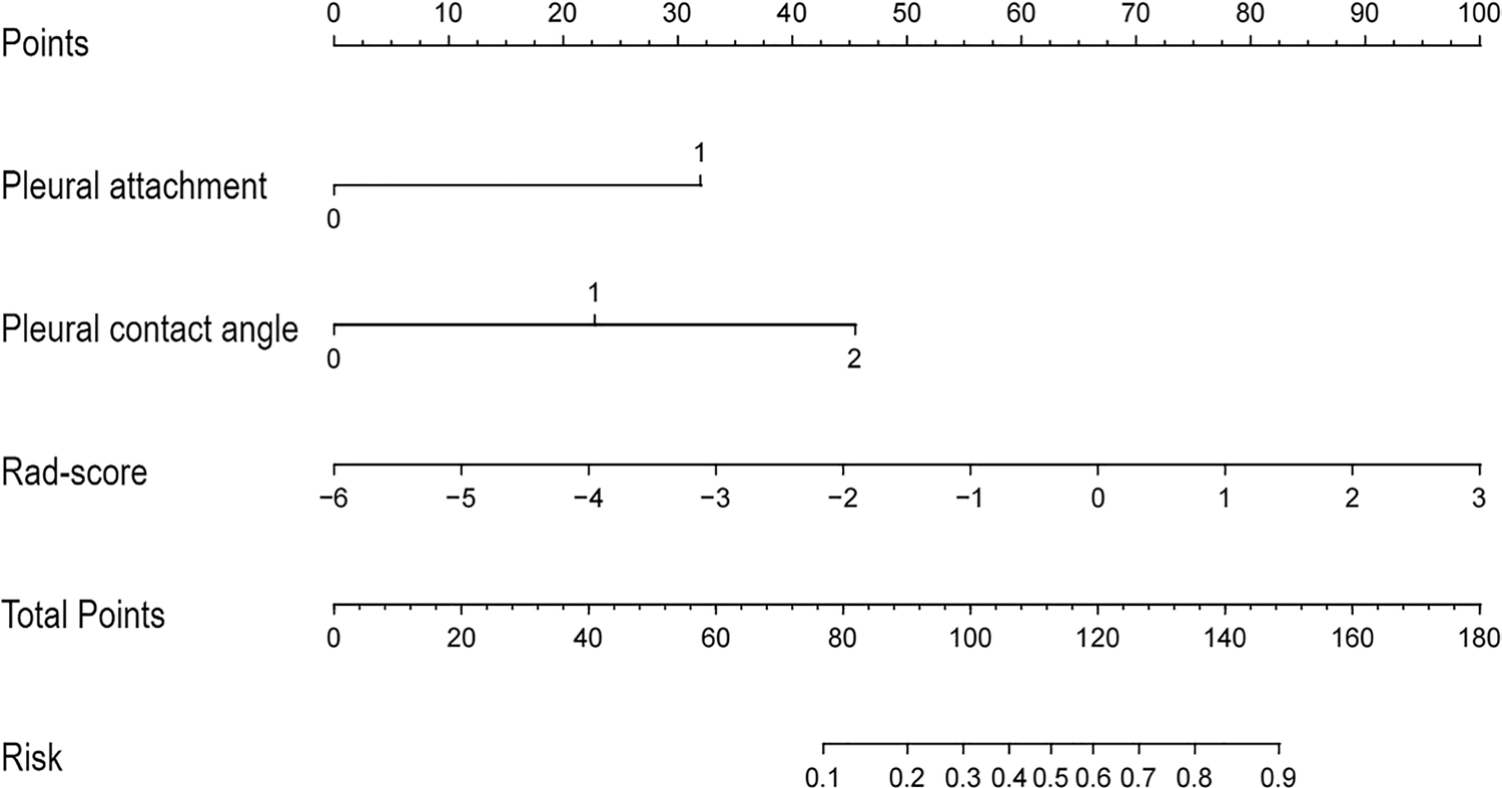
Nomogram predicting the likelihood of VPI in clinical stage Ia LA. According to the location of value on the second to the fourth axis, we can get the vertically corresponding points on the first axis. Summing up the three points together, we can get the total points and the vertically corresponding predicted value on the last axis

**Fig. 5:**
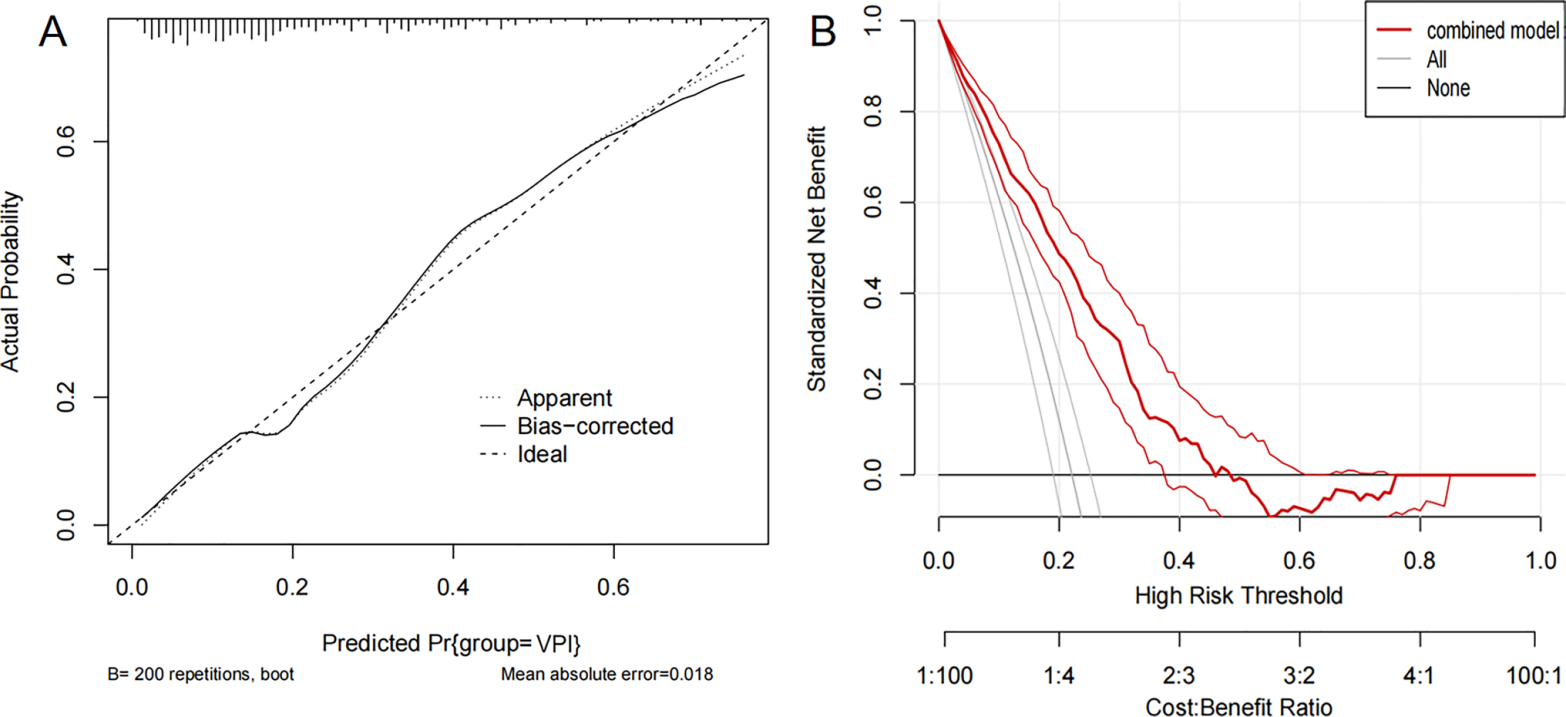
**A** Calibration curve of the logistic regression analysis based on the combined model (training group, n = 376). **B** Decision curve of the nomogram model for predicting the risk of VPI. The black line represents the assumption that no patients have VPI. The gray line represents the assumption that all patients have VPI. The red line represents the net benefit of using the nomogram model to predict VPI. The decision curve demonstrates that if the threshold probability ranges from 10 to 45%, using the nomogram for VPI prediction adds more benefit than predicting either all or no patients

The multivariable logistic regression analysis revealed that pleural attachment (OR = 3.28, 95% CI 1.85, 4.93, p < 0.001), larger pleural contact angle (OR = 1.88, 95% CI 1.31, 2.84, p = 0.019) and Rad-score (OR = 2.76, 95% CI 2.05, 3.64, p < 0.001) were significant independent predictors (Table 5). A nomogram integrating these predictors was constructed and displayed in Fig. 4. The outcome of the Hosmer–Lemeshow goodness-of-fit test yielded a non-significant result (p = 0.655), suggesting a strong agreement between the anticipated and actual probabilities. The calibration curve and DCA were depicted in Fig. 5. DCA confirmed clinical utility across 10–45% threshold probabilities, demonstrating superior net benefit versus ‘‘treat-all’’ and ‘‘treat-none’’ strategies.

**Table 5:** Multivariable logistic regression analysis of peritumoral semantic features combined with rad-score predicting the presence of VPI

Variable	Significant predictors		
	P Value	Odds ratio (95% CI)	Value in the formula*
Pleural attachment	< 0.001		0 or 1
0		Reference	
1		3.28 (1.85, 4.93)	
DLP	0.452		NA
Pleural contact angle	0.019		0 or 1
0		Reference	
1		Reference	
2		1.88 (1.31, 2.84)	
Rad-score	< 0.001	2.76 (2.05, 3.64)	Numeric value
EGFR	0.692		NA

VPI, visceral pleural invasion; Rad-score, radiomics score; CI, confidence interval; DLP, distance from the lesions to the adjacent pleura; EGFR, epidermal growth factor receptor; NA, not applicable (variables that were not included in the equation of multivariate logistic regression analysis with backward stepwise selection).

^*^Formula: e^x^/(1 + e^x^), x = − 0.813 + 0.621 × pleural attachment + 0.316 × Pleural contact angle (level 2) + 0.564 × Rad-score

**Fig. 6:**
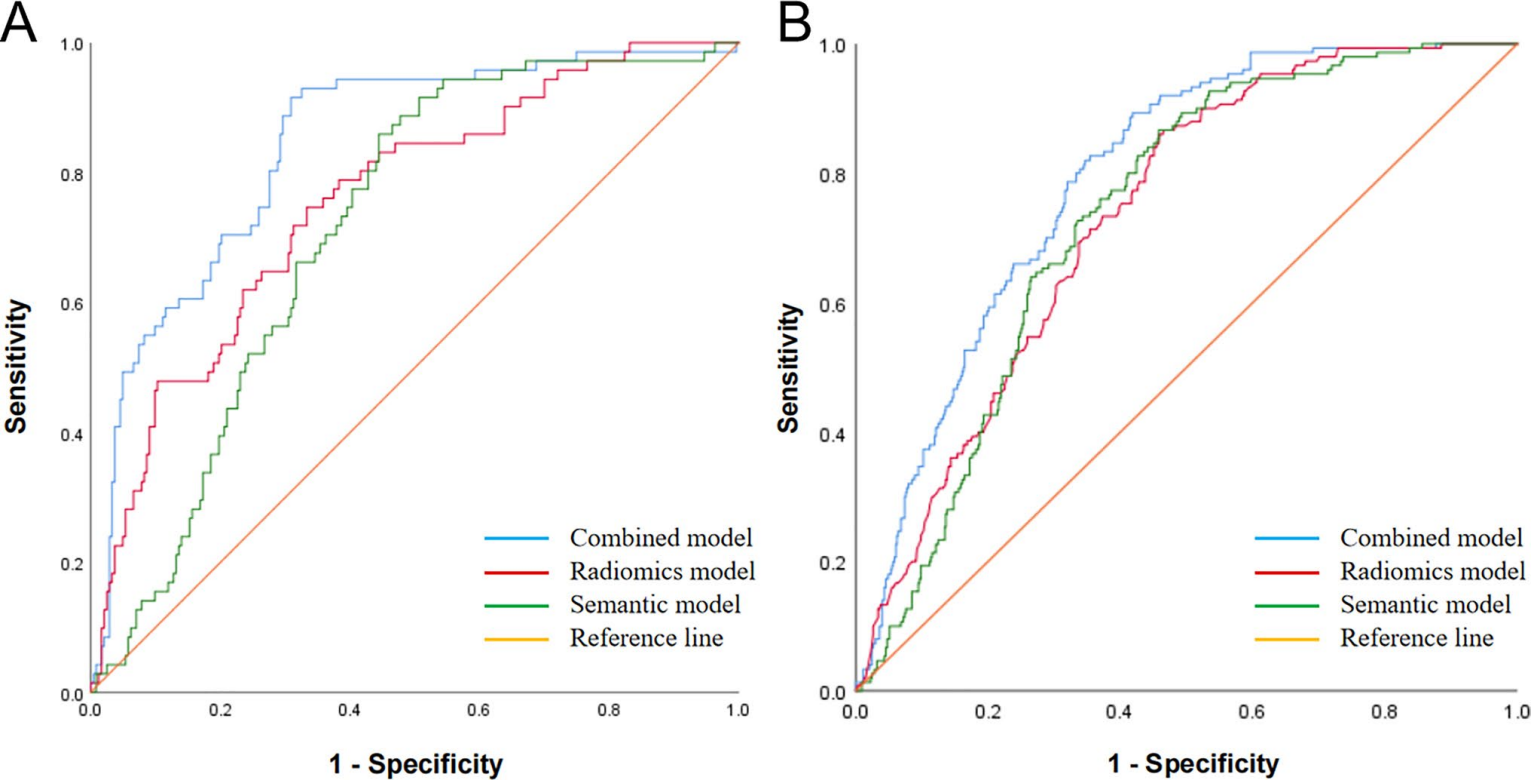
**A** The ROC curve for training group. The AUC of combined model increased to 0.843 (95% CI 0.796, 0.882), in contrast to 0.757 (95% CI 0.724, 0.785; DeLong’s test P < 0.001) and 0.715 (95% CI 0.688, 0.746; DeLong’s test P < 0.001) when only radiomics or CT semantic features were utilized separately. (B) The ROC curve for validation group. The AUC of combined model increased to 0.792 (95% CI: 0.765, 0.824), in contrast to 0.735 (95% CI 0.702, 0.769; DeLong’s test P < 0.001) and 0.733 (95% CI 0.696, 0.764; DeLong’s test P < 0.001) when only radiomics or CT semantic features were utilized separately

The AUC of combined model increased to 0.843 (95% CI 0.796, 0.882), in contrast to 0.757 (95% CI 0.724, 0.785; DeLong’s test P < 0.001) and 0.715 (95% CI 0.688, 0.746; DeLong’s test P < 0.001) when only radiomics or CT semantic features were utilized separately (Fig. 6A). The combined model exhibited a accuracy of 79.9%, precision of 61.3%, sensitivity of 83.5%, specificity of 78.5%, and F1-score of 70.7% when the cutoff was determined at the maximum Youden index. AUC of 0.792 (95% CI 0.765, 0.824) indicated a reasonable accuracy of combined model for the validation cohort with a accuracy of 72.0%, precision of 51.0%, sensitivity of 80.2%, specificity of 68.6%, and F1-score of 62.3% (Fig. 6B).

## Discussion

VPI has been regarded as an adverse prognostic factor in LA, correlating with increased recurrence risk and reduced overall survival, even in small lung neoplasms ≤ 2 cm or ground-glass opacity lesions [8, 14]. Kudo et al. proposed that pleural proximity facilitates rapid lymphatic spread through the visceral pleura's extensive lymphatic network, potentially explaining the broader spectrum of LN metastases observed in VPI-positive cases [15]. Accurate preoperative assessment for VPI is crucial for surgical planning and prognostic evaluation [16], yet current intraoperative frozen section analysis shows limited reliability (56.5% accuracy) [17], leaving postoperative elastic fiber staining as the diagnostic gold standard. The precise preoperative assessment in cases of subpleural lung cancer using imaging techniques may significantly influence surgical decision-making, a task that remains challenging. Subpleural LA typically manifests as pure GGNs that, due to their limited invasiveness, do not penetrate the visceral pleura (1/89 cases in our cohort) [18, 19]. Thus, the study population was limited to patients with peripheral pleural-associated LA at clinical stage Ia and excluding pure GGNs.

While previous CT studies have identified various VPI predictors including pleural contact, solid component > 50%, tumor size > 2 cm [20], consolidation-to-tumor ratio > 63% [21], and progressive risk elevation with increasing tumor size [22]. Our analysis revealed no significant associations between VPI and solid component proportions, maximum tumor diameter, or consolidation size. These discrepancies may stem from differences in inclusion criteria and potential selection biases, as our study specifically examined sub-3 cm peripheral LA while excluding pure GGNs and non-subpleural lesions. Notably, we found no correlation between conventional CT semantic features (spiculation, lobulation, air bronchogram) and VPI, consistent with prior reports questioning their predictive value [23–25]. While air bronchogram reflects non-destructive tumor growth along airways [26], and spiculation/lobulation indicate invasive growth patterns [27], these features appear insufficient for comprehensive VPI assessment, prompting our focus on peritumoral characteristics.

Our findings align with previous research emphasizing the predictive value of peritumoral radiological characteristics [28, 29]. The DLP, contact interface angle, and contact length showed significant associations with VPI. Hsu and Zhao et al. have underscored the potential utility of pleural attachment and indentation as valuable adjuncts for enhancing the early diagnostic accuracy of VPI [30, 31]. However, we observed no correlation with pleural indentation—a finding consistent with Gallagher's hypothesis that indentation reflects pleural tension from fibrotic changes rather than direct invasion [17, 32]. Multivariate analysis identified pleural attachment as the strongest VPI predictor, surpassing DLP in diagnostic performance due to the pleural invasion resulting from direct contact. While numerous studies [30, 33, 34] have investigated CT-based morphological features of VPI, their diagnostic accuracy remains constrained by dependence on radiologists’ expertise in feature interpretation.

The radiomics analysis focused on intratumoral features due to challenges in precisely delineating the ROI surrounding the subpleural tumor. Through Spearman correlation, mRMR, and LASSO regression, we identified five optimal radiomics features: total energy, skewness, large area low gray level emphasis (LALGLE), dependence non uniformity normalized (DNUN), and dependence entropy. These parameters systematically encompass features ranging from first-order statistics to higher-order texture descriptors [35]. Following multi-scanner harmonization via the ComBat algorithm and subsequent normalization through Z-score transformation, the resulting quantitative biomarkers exhibit satisfactory inter-observer agreement (ICC > 0.85) with cross-platform reproducibility demonstrating < 15% coefficient of variation.

Skewness and total energy are classified as first-order parameters, with lower values indicating greater lesion heterogeneity. LALGLE, a parameter of the GLSZM, also reflects lesion heterogeneity, with higher values indicating increased heterogeneity. The dependence entropy and DNUN, calculated from the GLDM, demonstrate the relationship between the gray-level intensity of CT voxels and the invasiveness of GGNs. A higher value of these features suggests increased heterogeneity in texture patterns, which indicating malignant trait of tumors, encompasses localized variances in tumor proliferation, metabolic activity, cell apoptosis, and blood supply [36]. The absence of shape-related features in our model parallels CT semantic findings, suggesting limited morphological distinction between VPI and non-VPI groups.

However, our study has certain limitations. It is important to note that this study is retrospective in nature, which may introduce selection bias. Additionally, variations in CT scanning devices and acquisition protocols could still impact the consistency of radiomics features, though image preprocessing and combat algorithm were performed. Furthermore, the feasibility and reproducibility of volume segmentation in clinical practice may be limited, with potential time constraints. Collaborative multi-center research is necessary to confirm the reliability and generalizability of the predictive model proposed in this study.

## Conclusion

This study presents a non-invasive predictive model combining tumor radiomics and peritumoral semantic features for preoperative VPI assessment in stage Ia peripheral LA. This decision-support tool could optimize surgical planning by identifying candidates for limited resection, potentially improving outcomes through personalized therapeutic strategies.

## Supplementary Information

Supplementary material 1

## Data Availability

The datasets used and/or analysed during the current study available from the corresponding author on reasonable request.

## References

[1] Siegel RL, Miller KD, Fuchs HE, Jemal A. Cancer statistics, 2022. CA Cancer J Clin. 2022;72(1):7–33. 35020204 10.3322/caac.21708

[2] Miller KD, Nogueira L, Devasia T, Mariotto AB, Yabroff KR, Jemal A, et al. Cancer treatment and survivorship statistics, 2022. CA Cancer J Clin. 2022;72(5):409–36. 35736631 10.3322/caac.21731

[3] Travis WD, Brambilla E, Rami-Porta R, Vallieres E, Tsuboi M, Rusch V, et al. International staging committee international staging C: visceral pleural invasion: pathologic criteria and use of elastic stains: proposal for the 7th edition of the TNM classification for lung cancer. J Thorac Oncol. 2008;2008(3):1384–90. 10.1097/JTO.0b013e31818e0d9f 19057261

[4] Butnor KJ, Travis WD. Recent advances in our understanding of lung cancer visceral pleural invasion and other forms of minimal invasion: implications for the next TNM classification. Eur J Cardiothorac Surg. 2013;43:309–11. 22843514 10.1093/ejcts/ezs429

[5] Rami-Porta R, Bolejack V, Crowley J, Ball D, Kim J, Lyons G, et al. The IASLC lung cancer staging project proposals for the revisions of the T descriptors in the forthcoming eighth edition of the TNM classification for lung cancer. J Thorac Oncol. 2015;10(7):990–1003. 26134221 10.1097/JTO.0000000000000559

[6] Zhang T, Zhang JT, Li WF, Lin JT, Liu SY, Yan HH, et al. Visceral pleural invasion in T1 tumors (</=3cm), particularly T1a, in the eighth tumor node-metastasis classification system for non-small cell lung cancer: a population-based study. J Thorac Dis. 2019;11:2754–62. 31463103 10.21037/jtd.2019.06.32 PMC6688036

[7] Huang Y, Liu Z, He L, Chen X, Pan D, Ma Z, et al. Radiomics signature: a potential biomarker for the prediction of disease-free survival in early-stage (I or II) non-small cell lung cancer. Radiology. 2016;281:947–57. 27347764 10.1148/radiol.2016152234

[8] Heidinger BH, Schwarz-Nemec U, Anderson KR, de MargerieMellon C, Monteiro Filho AC, Chen Y, et al. Visceral pleural invasion in pulmonary adenocarcinoma: differences in CT patterns between solid and subsolid cancers. Radiol Cardiothorac Imaging. 2019;1: e190071. 33778512 10.1148/ryct.2019190071 PMC7977962

[9] Fan L, Fang M, Li Z, Tu W, Wang S, Chen W, et al. Radiomics signature: a biomarker for the preoperative discrimination of lung invasive adenocarcinoma manifesting as a ground-glass nodule. Eur Radiol. 2019;29:889–97. 29967956 10.1007/s00330-018-5530-z

[10] Travis WD, Brambilla E, Nicholson AG, Yatabe Y, Austin JHM, Beasley MB, et al. The 2015 world health organization classification of lung tumors impact of genetic, clinical and radiologic advances since the 2004 classification. J Thorac Oncol. 2015;10(9):1243–60. 26291008 10.1097/JTO.0000000000000630

[11] Oyama M, Miyagi Maeshima A, Tochigi N, Tsuta K, Kawachi R, Sakurai H, et al. Prognostic impact of pleural invasion in 1488 patients with surgically resected non-small cell lung carcinoma. Jpn J Clin Oncol. 2013;43(5):540–6. 23487441 10.1093/jjco/hyt039

[12] Van Griethuysen JJM, Fedorov A, Parmar C, Hosny A, Aucoin N, Narayan V, et al. Computational radiomics system to decode the radiographic phenotype. Cancer Res. 2017;77(21):E104–7. 29092951 10.1158/0008-5472.CAN-17-0339 PMC5672828

[13] Orlhac F, Frouin F, Nioche C, Ayache N, Buvat I. Validation of a method to compensate multicenter effects affecting CT radiomics. Radiology. 2019;291(1):52–8. 10.1148/radiol.2019182023 30694160

[14] Zhang X, Xie J, Hu S, Peng W, Xu B, Li Y, et al. Prognostic value of visceral pleural invasion in the stage pT1-2N2M0 non-small cell lung cancer: a study based on the SEER registry. Curr Probl Cancer. 2021;45: 100640. 32828574 10.1016/j.currproblcancer.2020.100640

[15] Kudo Y, Saji H, Shimada Y, Nomura M, Matsubayashi J, Nagao T, et al. Impact of visceral pleural invasion on the survival of patients with non-small cell lung cancer. Lung Cancer. 2012;78:153–60. 22944144 10.1016/j.lungcan.2012.08.004

[16] Zhao LL, Xie HK, Zhang LP, Zha JY, Zhou FY, Jiang GN, et al. Visceral pleural invasion in lung adenocarcinoma </=3cm with ground-glass opacity: a clinical, pathological and radiological study. J Thorac Dis. 2016;8:1788–97. 27499970 10.21037/jtd.2016.05.90 PMC4958868

[17] Goldstraw P, Chansky K, Crowley J, Rami-Porta R, Asamura H, Eberhardt WEE, et al. The IASLC lung cancer staging project: proposals or revision of the TNM stage groupings in the forthcoming (Eighth) edition of the TNM classification for lung cancer. J Thorac Oncol. 2016;11(1):39–51. 26762738 10.1016/j.jtho.2015.09.009

[18] Zhao Q, Wang JW, Yang L, Xue LY, Lu WW. CT diagnosis of pleural and stromal invasion in malignant subpleural pure ground-glass nodules: an exploratory study. Eur Radiol. 2019;29(1):279–86. 29943186 10.1007/s00330-018-5558-0

[19] Shi J, Li F, Yang F, Dong Z, Jiang Y, Nachira D, et al. The combination of computed tomography features and circulating tumor cells increases the surgical prediction of visceral pleural invasion in clinical T1N0M0 lung adenocarcinoma. Transl Lung Cancer Res. 2021;10(11):4266–80. 35004255 10.21037/tlcr-21-896 PMC8674597

[20] Seok Y, Lee E. Visceral pleural invasion is a significant prognostic factor in patients with partly solid lung adenocarcinoma sized 30 mm or smaller. Thorac Cardiovasc Surg. 2018;66(2):150–5. 27517168 10.1055/s-0036-1586757

[21] Yanagawa M, Tanaka Y, Leung AN, Morii E, Kusumoto M, Watanabe S, et al. Prognostic importance of volumetric measurements in stage I lung adenocarcinoma. Radiology. 2014;272(2):557–67. 24708191 10.1148/radiol.14131903

[22] Manac’h D, Riquet M, Medioni J, Le Pimpec-Barthes F, Dujon A, Danel C, et al. Visceral pleura invasion by nonsmall cell lung cancer: an underrated bad prognostic factor. Ann Thorac Surg. 2001;71(4):1088–93. 11308141 10.1016/s0003-4975(00)02649-7

[23] Ahn SY, Park CM, Jeon YK, Kim H, Lee JH, Hwang EJ, et al. Predictive CT features of visceral pleural invasion by T1-sized peripheral pulmonary adenocarcinomas manifesting as subsolid nodules. AJR Am J Roentgenol. 2017;209:561–6. 28639833 10.2214/AJR.16.17280

[24] Deng HY, Li G, Luo J, Alai G, Zhuo ZG, Lin YD. Novel biologic factors correlated to visceral pleural invasion in early-stage non-small cell lung cancer less than 3 cm. J Thorac Dis. 2018;10(4):2357–64. 29850141 10.21037/jtd.2018.03.185 PMC5949454

[25] Wang F, Pan XL, Zhang T, Zhong Y, Wang CL, Li H, et al. Predicting visceral pleural invasion in lung adenocarcinoma presenting as part-solid density utilizing a nomogram model combined with radiomics and clinical features. Thorac Cancer. 2024;15(1):23–34. 38018018 10.1111/1759-7714.15151 PMC10761615

[26] Lederlin M, Puderbach M, Muley T, Schnabel PA, Stenzinger A, Kauczor HU, et al. Correlation of radio- and histomorphological pattern of pulmonary adenocarcinoma. Eur Respir J. 2013;41:943–51. 22835610 10.1183/09031936.00056612

[27] Wang Y, Lyu D, Zhou TH, Tu WT, Fan L, Liu SY. Multivariate analysis based on the maximum standard unit value of ^18^F-fluorodeoxyglucose positron emission tomography/computed tomography and computed tomography features for preoperative predicting of visceral pleural invasion in patients with subpleural clinical stage IA peripheral lung adenocarcinoma. Diagn Interv Radiol. 2023;29(2):379–89. 36988049 10.4274/dir.2023.222006 PMC10679694

[28] Glazer HS, Duncan-Meyer J, Aronberg DJ, Moran JF, Levitt RG, Sagel SS, et al. Pleural and chest wall invasion in bronchogenic carcinoma: CT evaluation. Radiology. 1985;157(1):191–4. 4034965 10.1148/radiology.157.1.4034965

[29] Chen Z, Jiang S, Li Z, Rao L, Zhang X. Clinical value of ^18^F-FDG PET/CT in prediction of visceral pleural invasion of subsolid nodule stage I lung adenocarcinoma. Acad Radiol. 2020;27(12):1691–9. 32063495 10.1016/j.acra.2020.01.019

[30] Hsu JS, Han I-T, Tsai TH, Lin SF, Jaw TS, Liu GC, et al. Pleural tags on CT scans to predict visceral pleural invasion of non–small cell lung cancer that does not abut the pleura. Radiology. 2016;279(2):590–6. 26653684 10.1148/radiol.2015151120

[31] Gruden JF. What is the significance of pleural tags? AJR Am J Roentgenol. 1995;164(2):503–4. 7840000 10.2214/ajr.164.2.7840000

[32] Gallagher B, Urbanski SJ. The significance of pleural elastica invasion by lung carcinomas. Hum Pathol. 1990;21(5):512–7. 1692562 10.1016/0046-8177(90)90007-r

[33] Ebara K, Takashima S, Jiang B, Numasaki H, Fujino M, Tomita Y, et al. Pleural invasion by peripheral lung cancer: prediction with three dimensional CT. Acad Radiol. 2015;22:310–9. 25542401 10.1016/j.acra.2014.10.002

[34] Gillies RJ, Kinahan PE, Hricak H. Radiomics: images are more than pictures. They Are Data Radiology. 2016;278:563–77. 26579733 10.1148/radiol.2015151169 PMC4734157

[35] Wei SH, Zhang JM, Shi B, Gao F, Zhang ZX, Qian LT. The value of CT radiomics features to predict visceral pleural invasion in ≤3 cm peripheral type early non-small cell lung cancer. J Xray Sci Technol. 2022;30(6):1115–26. 35938237 10.3233/XST-221220

[36] Nelson DA, Tan TT, Rabson AB, Anderson D, Degenhardt K, White E. Hypoxia and defective apoptosis drive genomic instability and tumorigenesis. Genes Dev. 2004;18:2095–107. 15314031 10.1101/gad.1204904 PMC515288

