# Supplementary material for: Integrating radiomics features and CT semantic characteristics for predicting visceral pleural invasion in clinical stage Ia peripheral lung adenocarcinoma
Source: Discov Oncol. 2025 May 16;16:780. doi: 10.1007/s12672-025-02548-6 (PMC12084461; doi:10.1007/s12672-025-02548-6)
Supplement: Supplementary file 1 — Supplementary material 1 [file 12672_2025_2548_MOESM1_ESM.docx]

| **Table S1.** Agreement of the key Radiomics Features between the Two Readers and the CV between Different Scanners. | | | | |
| --- | --- | --- | --- | --- |
| **Radiomics Features** | **ICC** | **95% CI** | **CV before ComBat (%)** | **CV after ComBat (%)** |
| **original_glszm_Large Area Low Gray Level Emphasis**  **exponential_firstorder_Total Energy**  exponential_gldm_Small Dependence High Gray Level Emphasis  exponential_glrlm_Run Length Non Uniformity  **square_gldm_Dependence Non Uniformity Normalized**  square_glszm_Small Area Low Gray Level Emphasis  squareroot_firstorder_Skewness  wavelet-LLH_glcm_MCC  wavelet-LLH_ngtdm_Busyness  **wavelet-LHL_firstorder_Skewness**  wavelet-HLL_glszm_Large Area Low Gray Level Emphasis  **wavelet-LLL_gldm_Dependence Entropy** | 0.91  0.95  0.79  0.85  0.89  0.90  0.81  0.87  0.84  0.90  0.85  0.87 | 0.87, 0.95  0.92, 0.97  0.71, 0.86  0.83, 0.88  0.82, 0.95  0.85, 0.94  0.78, 0.85  0.80, 0.93  0.80, 0.88  0.88, 0.93  0.78, 0.91  0.84, 0.90 | 22.5  11.7  14.2  16.4  21.3  15.2  7.6  16.0  9.7  10.6  20.1  13.8 | 14.5  8.6  11.6  10.7  13.9  9.5  5.6  11.4  7.6  8.8  14.3  11.4 |
| CV, coefficient of variation; ICC, intraclass correlation coefficient; CI, confidence interval. | | | | |

| **Table S2.** Agreement of CT Semantic Features between the Two Readers. | | |
| --- | --- | --- |
| **CT Features** | **Value** | **Kendall Coefficient of Concordance** |
| Contour | 0.89 | 0.91 |
| Lobulation | 0.90 | 0.91 |
| Spiculation | 0.93 | 0.92 |
| Texture | 0.92 | 0.94 |
| Calcification | 0.99 | NA |
| Air bronchogram | 0.94 | 0.95 |
| Bubble-like lucency | 0.93 | 0.94 |
| Cavity | 0.96 | 0.99 |
| Pleural attachment | 0.99 | NA |
| Pleural indentation | 0.96 | NA |
| Pleural contact angle | 0.94 | 0.95 |
| Bronchovascular bundle thickening | 0.87 | 0.89 |
| Obstructive change | 0.97 | NA |
| Pleural effusion of tumor side | 0.99 | NA |
| Pleural effusion of non-tumor side | 0.99 | NA |
| CT, computed tomography; NA, not applicable | | |

| **Table S3.** Association of Clinical Characteristics between Training Group and Validation Group. | | | |
| --- | --- | --- | --- |
| **Variable** | **Training group** | **Validation**  **group** | **P Value** |
| Number  Age(years)  Sex  Male  Female  Smoking history  Yes  No  Family history  Yes  No  Histological subtype  Low risk  Moderate risk  High risk  EGFR  Mutation  Wild  Pathological stage  I  II  III  VPI  Presence  Absence | 376  59.48(±8.65)  148  228  152  224  87  289  31  159  186  132  63  332  13  31  32  344 | 161  57.84(±8.13)  65  96  73  88  42  119  17  58  86  58  31  138  7  16  18  143 | 0.375  0.826  0.290  0.464  0.615  0.675  0.410  0.329 |
| Data for age is mean ± standard deviation.  EGFR, epidermal growth factor receptor; VPI, visceral pleural invasion. | | | |

| **Table S4.** Association between CT semantic features of primary tumor with VPI in Training Group. | | | |
| --- | --- | --- | --- |
| **Variable** | **Negative Group**  **(n=344)** | **VPI Group**  **(n=32)** | **P Value** |
| Contour |  |  |  |
| 1 | 28 | 3 | 0.869 |
| 2 | 185 | 16 |  |
| 3 | 131 | 13 |  |
| Maximum diameter (cm) | 2.22 (±0.65) | 2.24 (±0.62) | 0.776 |
| Consolidation diameter (cm) | 1.94 (±0.69) | 1.96 (±0.62) | 0.970 |
| TDR | 0.13 (±0.19) | 0.11 (±0.19) | 0.589 |
| Calcification |  |  |  |
| 0 | 333 | 32 | 0.609 |
| 1 | 11 | 0 |  |
| Air bronchogram |  |  |  |
| 0 | 212 | 23 | 0.252 |
| 1 | 132 | 9 |  |
| Bubble-like lucency |  |  |  |
| 0 | 315 | 31 | 0.473 |
| 1 | 29 | 1 |  |
| Cavity |  |  |  |
| 0 | 328 | 30 | 1.000 |
| 1 | 16 | 2 |  |
| Texture |  |  |  |
| 1 | 99 | 6 | 0.148 |
| 2 | 74 | 6 |  |
| 3 | 171 | 20 |  |
| Lobulation |  |  |  |
| 0 | 203 | 15 | 0.183 |
| 1 | 141 | 17 |  |
| Spiculation |  |  |  |
| 0 | 270 | 23 | 0.388 |
| 1 | 74 | 9 |  |
| Data for maximum diameter, consolidation diameter and TDR are mean ± standard deviation.  CT, computed tomography; VPI, visceral pleural invasion; TDR, tumor shadow disappear rate. | | | |
